# Optimization Design of Asphalt Mixture Composite Reinforced with Calcium Sulfate Anhydrous Whisker and Polyester Fiber Based on Response Surface Methodology

**DOI:** 10.3390/ma16020594

**Published:** 2023-01-07

**Authors:** Taotao Fan, Chundi Si, Yi Zhang, Yuefeng Zhu, Song Li

**Affiliations:** 1 School of Traffic and Transportation, Shijiazhuang Tiedao University, Shijiazhuang 050043, China; 2 State Key Laboratory of Mechanical Behavior and System Safety of Traffic Engineering Structures, Shijiazhuang Tiedao University, Shijiazhuang 050043, China

**Keywords:** asphalt mixture, calcium sulfate anhydrous whisker, polyester fiber, central composite circumscribed design, design parameters optimization, low-temperature behavior

## Abstract

In order to improve the properties of calcium sulfate anhydrous whisker (ACSW) and polyester fiber composite reinforced asphalt mixture (ACPRA) to meet the service requirements of pavement materials in low-temperature environments, the central composite circumscribed design (CCC), a kind of response surface methodology, was chosen to optimize the design parameters. Three independence variables, asphalt aggregate ratio, ACSW content, and polyester fiber content were adopted to evaluate the design parameters. Four responsive variables, air voids, Marshall stability, splitting tensile strength, and failure tensile strain, were chosen to study the volumetric and mechanical characteristics, and the low-temperature behavior of ACPRA by the Marshall test and indirect tensile test at −10 °C. The results showed that, taking low-temperature behavior optimization as the objective, the CCC method was practicable to optimize design of ACPRA, and the optimization design parameters were asphalt aggregate ratio of 4.0%, ACSW content of 10.8%, and polyester fiber content of 0.4%. Furthermore, the impact of three independence variables interactions on four response variables was also discussed, and it was identified that the interaction between asphalt aggregate ratio and ACSW content, and between asphalt aggregate ratio and polyester fiber content, has greater bearing on the splitting tensile strength and failure tensile strain of APCRA. Meanwhile, ACSW and polyester fiber enhancing the low-temperature behavior of APCRA was primarily connected with their contents.

## 1. Introduction

In vehicle load and low-temperature cases, the tensile ability of pavement materials is negative, which results in the dehiscence of asphalt pavement. For this reason, there is a need to enhance the tensile ability of pavement materials for extending the service time of pavements. 

Nowadays, fiber-reinforced asphalt mixture has been popularized and applied in road engineering. The selected fiber modifier includes polyester fiber, basalt fiber, steel fiber, etc. [1,2,3,4]. Previous research suggests that fiber modifier can strengthen the low-temperature anti-crack and fatigue behavior of asphalt mixture [5,6,7,8]. The results of J.R. Wu et al. evaluated that the improvement impact of polyester fiber on low-temperature anti-fracture asphalt mixture makes it able to resist I-II compound cracks [9,10]. Y.P. Sheng et al. discovered that polyester fiber would enhance the anti-crack and water damage resistibility of asphalt mixture [11]. Q.W. Xu et al. evaluated that polyester fiber could enhance the flexural strength and the indirect tensile strength of asphalt concrete [12]. 

Calcium sulfate whisker (CSW) is a kind of solid waste recycling material and is mainly prepared from phosphogysum and flue gas desulfurization gypsum [13,14]. Based on the water of crystallization, there are three types of CSW, calcium sulfate dehydrates whisker, calcium sulfate hemihydrates whisker, and calcium sulfate anhydrous whisker (ACSW). In addition, as an eco-friendly material, CSW is widely used in a range of applications in terms of combined materials [15,16], building materials [17,18], and fireproofing [19,20] due to the merit of its high modulus and thermostability. In pavement engineering, J.H. Ma found that CSW with surface modification has good consistency with asphalt, and the softening point value increased as CSW dosage rose, while the low temperature of asphalt was substantially reduced by mixing with surface-modified or unmodified CSW [21]. G.Y. Li et al. found that when ACSW contents changed from 0 wt.% to 8 wt.%, the temperature sensitivity of the asphalt binder was negative, while its high-temperature and viscosity-temperature characterizations were raised [22]. T.T. Fan et al. investigated that when the content of CSW ranged from 7 wt.% to 11 wt.%, CSW could obviously raise the anti-deformation and rheological characterization of asphalt binder, while decreasing its thermo-sensitivity and low-temperature performance [23,24].

The traditional methodology of fiber-reinforced asphalt mixture usually designs the optimum dosage of asphalt through the Marshall test method at first, and then obtains the optimum fiber content in terms of performance test results. This methodology usually needs a lot of tests, resulting in a waste of time and resources [25]. Consequently, for the sake of obtaining the optimal design parameters of fiber-reinforced asphalt mixture in practice, it is important to adopt scientific testing methods to examine the relationship between design parameters and the characteristics of asphalt mixture.

Response surface methodology (RSM) is a statistical test method of optimizing stochastic procedure, which sets up a succession surface model, assesses the variables impacting the test process and their inter-reactions, and ascertains the best levels. Furthermore, this way saves laboratory time and reduces the manpower and material consumption. It is widely employed in chemical industry [26,27], materials science [28], building materials engineering [29], and pavement engineering [30,31,32]. Ahmed I. Nassar et al. applied RSM to optimize the mix proportion of emulsified asphalt mixtures [33]. Nura Bala et al. applied RSM to put forward a design of nano-silica-modified asphalt concrete based on the volumetric and engineering properties [34]. Zh.J. Tang et al. applied RSM to investigate separately the effect of shearing time, fiber dosage, and asphalt aggregate ratio on the moisture sensitivity of waste betel-nut-fiber-enhanced asphalt mixtures [35]. In total, there is an underlying advantage of using RSM as a replaceable method for the design parameter majorization of ACSW and polyester-fiber-composite-reinforced asphalt mixture (APCRA).

In the present study, RSM was adopted for experimental design to set up the response functions of APCRA. The asphalt aggregate ratio, ACSW content, and polyester fiber content were taken as independent variables, and the air voids, Marshall stability, splitting tensile strength, and failure tensile stain were taken as response variables. The impact of three independent variables on the four response variables of APCRA was analyzed, and its optimization design parameters with better low-temperature performance were obtained. 

## 2. Materials and Methods

### 2.1. Materials

In this paper, SK-90# asphalt binder (penetration grade: 80/100) was selected, and its basic physical characteristics are listed in Table 1.

The coarse and fine aggregates and mineral powder were obtained from a stone quarry in Xianyang of Shaanxi Province, China. Their physical characteristics were measured based on the Chinese standard JTG E42-2005 and separately given in Table 2 and Table 3.

**Figure 1:**
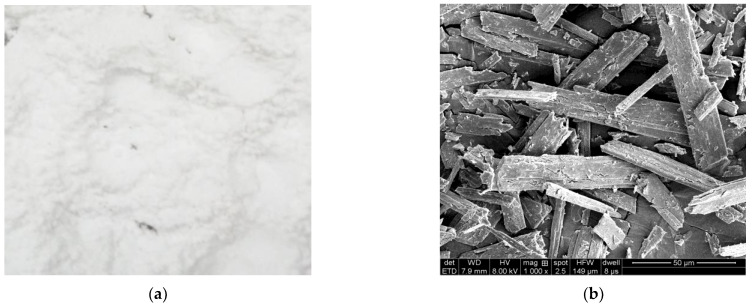
Morphology of ACSW: (**a**) macro-morphology, and (**b**) micro-morphology.

**Figure 2:**
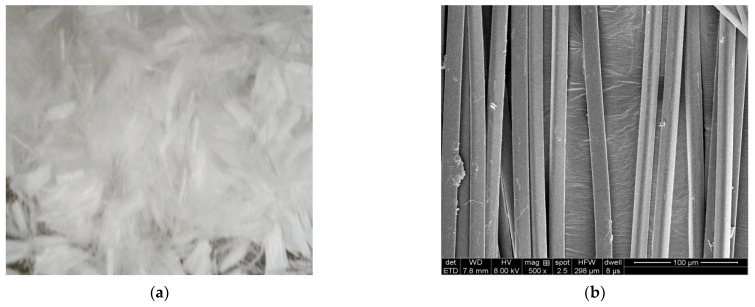
Morphology of polyester fiber: (**a**) macro-morphology, and (**b**) micro-morphology.

The physical characteristics of ACSW and polyester fiber are separately given in Table 4 and Table 5. The morphology of ACSW and polyester fiber are shown in Figure 1 and Figure 2, respectively.

### 2.2. Sample Preparation of APCRA

**Figure 3:**
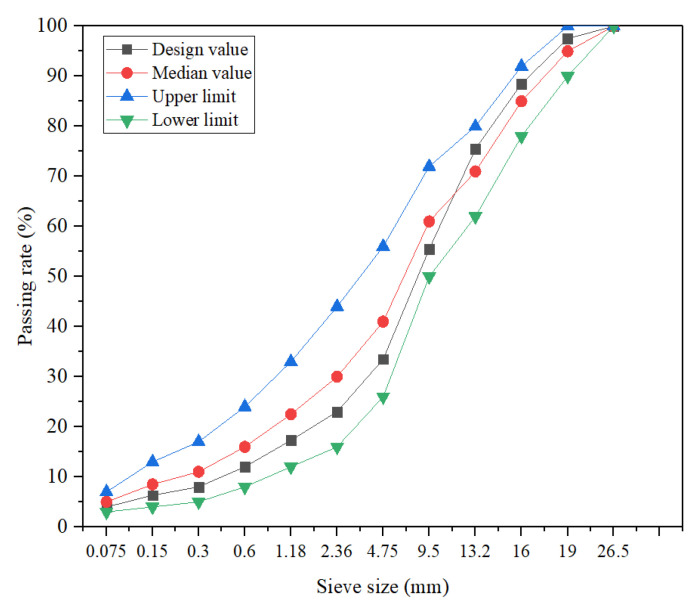
Gradation of AC-20.

Asphalt concrete samples were manufactured to explore the proportion design and optimization of APCRA. Figure 3 displays the gradation of asphalt concrete (AC) with a nominal maximum particle size of 19 mm. In accordance with the Chinese standard JTG E20-2011, the Marshall samples of AC with a height of 63.5 mm and a diameter of 101.6 mm were prepared using Marshall process for indoor laboratory and majorization design with RSM. APCRA was prepared in four steps: (1) the aggregates and mineral powder were weighted and put into an oven at 175 ± 5 °C for 2 h and base asphalt binder was put on 155 °C; (2) the weighted aggregates were poured into the mixing plant and mixed evenly, and then SK-90# asphalt binder was added and stirred at 170–175 °C until the aggregate surfaces were coated with asphalt binder; (3) the weighted ACSW, polyester fiber, and mineral powder were added and mixed well at 170–175 °C; (4) the mixed APCRAs were compacted 75 times on both sides at 150–155 °C.

### 2.3. Testing Methods

#### 2.3.1. Marshall Test 

Marshall stability (*MS*) and air voids (*AV*) of APCRA samples were measured by using Marshall test. In detail, before testing, APCRA samples were submerged at 60 °C for 30 min, and then following JTG E20-2011, an invariable vertical displacement speed of 50 mm/min was applied on APCRA samples until it was damaged.

The maximum load is defined as Marshall stability. The air voids can be calculated by Equation (1).

(1) $$AV=\left(1-\gamma_{f}/\gamma_{tmd}\right)\times 100$$

where γ*_f_* means the bulk volume relative density; γ*_tmd_* means the maximum theoretical relative density.

#### 2.3.2. Indirect Tensile Test

The low-temperature anti-crack of asphalt mixture was studied through indirect tensile test (IDT). In IDT, The Marshall samples bear the compression load between two loading spans (with a width of 12.7 mm) at an invariable vertical displacement speed. The loading rate of 1 mm/min was applied under −10 °C. The IDT was conducted using the Material Test System (equipment model: LETRY). In detail, the APCRA sample generates relatively uniform tensile stress along the vertical plane. With the growth of the applied vertical displacement, the tensile stress of the APCRA sample increases until it cracks along the vertical direction. The splitting tensile strength (*STS*) and failure tensile stain (*FTS*) were calculated separately according to Equations (2)–(4):

(2) $$STS=\frac{2\times P_{\max}}{\pi \times D\times H}$$

where *P*_max_ means the maximum load (kN); *D* and *H* mean the sample diameter and thickness, respectively (mm).

(3) $$FTS=\frac{X_{T}\times (0.0307+0.0936\mu )}{\left(1.35+5\mu \right)}$$

(4) $$X_{T}=\frac{Y_{T}\times (0.135+5\mu )}{\left(1.794-0.0314\mu \right)}$$

where *μ* means poisson’s ratio; *X_T_* and *Y_T_* mean the deformation in *X*-direction and *Y*-direction at damage load (mm), respectively.

### 2.4. Design of Experiments

**Figure 4:**
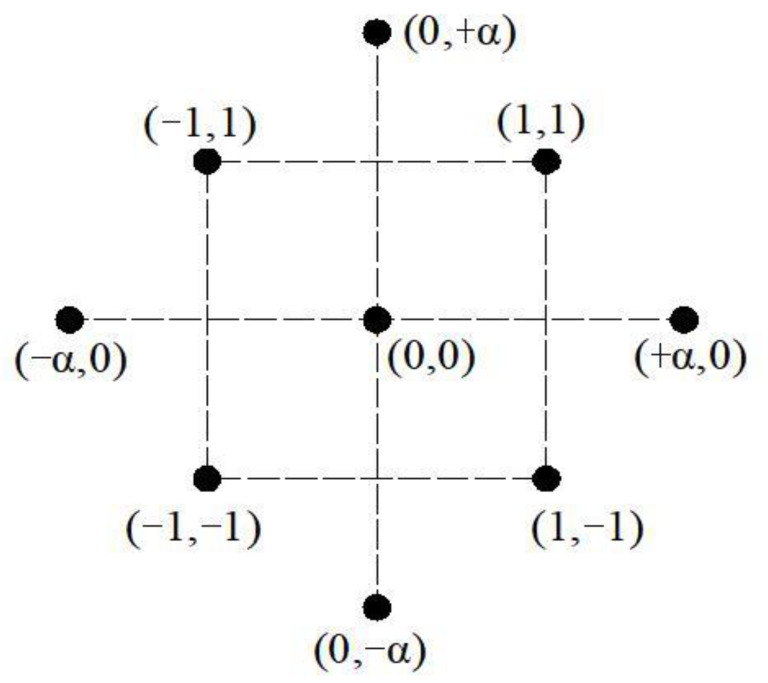
The horizontal point distribution of CCD.

Among the RSM techniques, central composite design (CCD) is an extensively adopted and effective method to statistically assess the inter-reaction between independent variables and response variables within the scope of an experiment. In CCD technology, at least two digital inputs are required and changed with the scope of alpha (*α*) through three (−1, 0, +1) or five (−*α*, −1, 0, +1, +*α*) phases, as shown in Figure 4. According to the value of *α*, the CCD model has three categories: central composite circumscribed design (CCC) (*α* > 1), central composite face centered design (*α* = 1), and central composite inscribed design (*α* < 1). In this study, CCC was chosen because of the advantage of considering both the sequence and the rotation in experiment design. The magnitude of experimental samples is calculated by (2*^i^* + 2*i* + n), wherein *i* means the amount of independent variables, *n* means the amount of central points. The value of *α* can be calculated by 2*^i^*^/4^.

In this paper, twenty experiments were devised by CCC, consisting of eight factorial points, six star points, and six central points at five experimental levels (*i* = 3, *n* = 5, *α* = 1.682). Central points were set to six because repeated tests were required to eliminate experimental errors. Three independent variables are asphalt aggregate ratio (the percentage of asphalt binder to aggregate mass ratio), ACSW content (the percentage of ACSW to asphalt binder mass ratio), and polyester fiber content (the percentage of polyester fiber to asphalt concrete mass ratio), which individually abbreviate to *AAR*(*X*_1_), *ACC* (*X*_2_), and *PFC*(*X*_3_), respectively. Based on previous research [23], the appropriate ACSW content changes from 7% to 13% of asphalt binder mass, and the appropriate polyester fiber content should exceed 0.4% of asphalt concrete mass. The optimum *AAR* content of base AC-20 mixtures was 4.1% by Marshall test method. The suitable scopes of *AAR*, *ACC,* and *PFC* were selected as listed in Table 6.

Four response variables, *AV*(*Y*_1_), *MS*(*Y*_2_), *TST*(*Y*_3_), and *FTS*(*Y*_4_), were used to optimize the experimental processes. The experimental design and results are shown in Table 7.

The Design-Expert 10.0 software was chosen to run the experimental program, complete the analysis of response model and statistical regression, and select the best combination of independent variable levels. Comparing four kinds of response models, linear, two-factor interaction, quadratic and cubic polynomials, the quadratic model shown in Equation (5) was proposed by document [30] to forecast the change rule of response variables.

(5) $$Y=\beta_{0}+\sum_{i=1}^{3}\beta_{i}X_{i}+\sum_{i=1}^{2}\sum_{j=i+1}^{3}\beta_{ij}X_{i}X_{j}+\sum_{i=1}^{3}\beta_{ii}X_{i}^{2}+\varepsilon$$

where *Y* means the responsive variable; *X_i_* and *X_j_* mean the coded independent parameters; *β*_0_, *β_i_*, *β_ij_*, and *β_ii_*, respectively mean the coefficient of terms of constant, primary, interaction, and quadratic; *ε* means the random error.

## 3. Results and Discussion

### 3.1. Statistical Modeling

Analysis of variance (ANOVA) was fulfilled to research the inter-reaction between different independent variables and their impacts on the response variables. The confidence level was 95%, that is, the model and independent variable were significant when the *p*-value was lower than 0.05. ANOVA results are separately listed in Table 8 and Table 9.

The ANOVA results for the quadratic model of *Y*_1_ (*AV*) are shown in the first row of Table 8. *R*-squared of 0.9943 and Adj. *R*-squared of 0.9893 implied that the *AV* model was well fitted. In addition, Adeq. precision was adopted to evaluate the signal-to-noise ratio of the response model, and the regression model is ideal when the value of Adeq. precision was larger than 4. From Table 8, the Adeq. precision of the *AV* model was 48.525, which indicated that the *AV* model was also well fitted. The model *p*-value of <0.0001 for *AV* declared that the *AV* model was significant.

Similarly, the ANOVA results in Table 8 indicated that the models of *MS*, *STS*, and *FTS* were also well fitted. 

From Table 9, it could be testified that at 95% confidence level (*p*-value < 0.05), the significant terms of the *AV* model included *X*_1_, *X*_3_, *X*_2_*X*_3_, (*X*_2_)^2^, and (*X*_3_)^2^. Likewise, the significant terms of the *MS* model were *X*_1_, *X*_3_, *X*_1_*X*_2_, *X*_1_*X*_3_, (*X*_1_)^2^, (*X*_2_)^2^, and (*X*_3_)^2^, that of the *STS* model were *X*_1_, *X*_1_*X*_2_, *X*_1_*X*_3_, *X*_2_*X*_3_, (*X*_1_)^2^, and (*X*_2_)^2^, and that of the *FTS* model were *X*_2_, *X*_3_, *X*_1_*X*_2_, *X*_1_*X*_3_, and (*X*_3_)^2^.

The significant of lack-of-fit is chosen to study the model fit. The *p*-values of lack-of-fit of *AV*, *MS*, *STS*, and *FTS* listed in Table 9 were 0.4538,0.1156,0.1816, and 0.1142, respectively, which manifested that all models were insignificant, namely that the models of *AV*, *MS*, *STS*, and *FTS* were well fitted.

After removing the insignificant terms, the equation between the design parameters and the properties responses of APCRA were posed as Equations (6)–(9):

(6) $$Y_{1}=4.78-2.03X_{1}+18.94X_{3}-0.43X_{2}X_{3}-0.02X_{2}^{2}-11.58X_{3}^{2}$$

(7) $$Y_{2}=-35.43+18.1X_{1}+1.91X_{3}-0.11X_{1}X_{2}-2.85X_{1}X_{3}-1.99X_{1}^{2}-0.06X_{2}^{2}+10.06X_{3}^{2}$$

(8) $$Y_{3}=-18.01+6.94X_{1}+0.14X_{1}X_{2}-4.85X_{1}X_{3}-0.49X_{2}X_{3}-0.80X_{1}^{2}-0.04X_{2}^{2}$$

(9) $$Y_{4}=-769.65+375.69X_{2}+6036.13X_{3}-102.41X_{1}X_{2}+1902.08X_{1}X_{3}-15240.73X_{3}^{2}$$

### 3.2. Diagnostics Analyses

**Figure 5:**
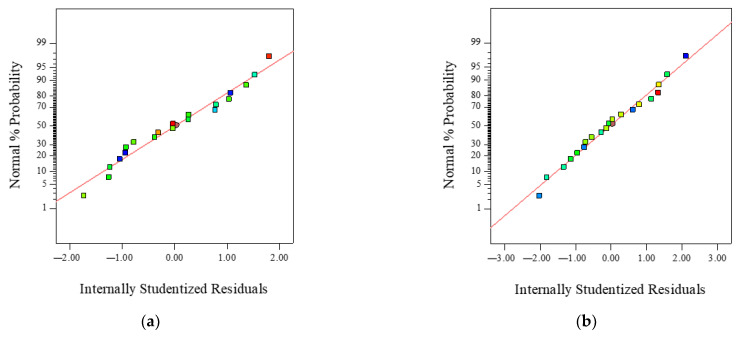

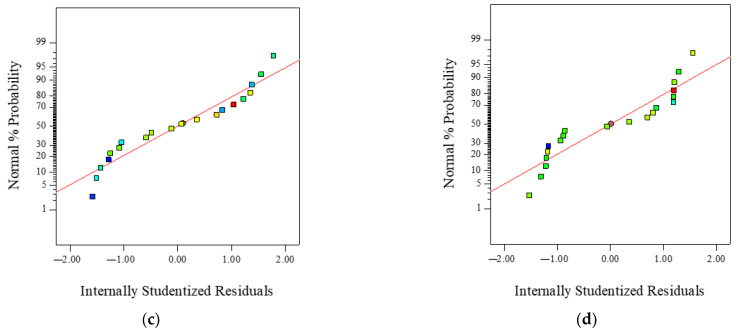
Diagnostics of responsive model: (**a**) *AV*; (**b**) *MS*; (**c**) *STS*; and (**d**) *FTS*.

To better realize model satisfaction, the normal probability of internally studentized residuals of four response models were shown in Figure 5.

From Figure 5, all data points were distributed approximately linearly, which implied the four response models are significant. In addition, the more obvious the linear distribution, the more significant the model, and this conclusion was testified by larger *R*-squared and Adj. *R*-squared of four response models (see Table 8).

### 3.3. Analysis of Response Surfaces

The concavity and convexity of the response surface and the contours shape are adopted to evaluate the impact of independent variable inter-reactions on the response variables [36], and the more convex the response surface and the closer the contour shape is to the saddle shape (or ellipse shape), the more obvious the inter-reaction.

#### 3.3.1. Analysis of AV

**Figure 6:**
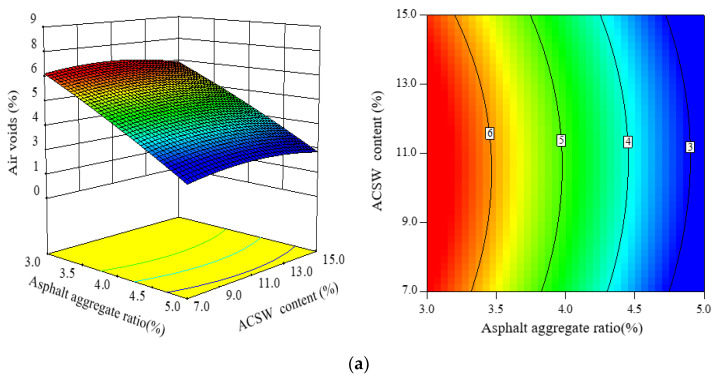

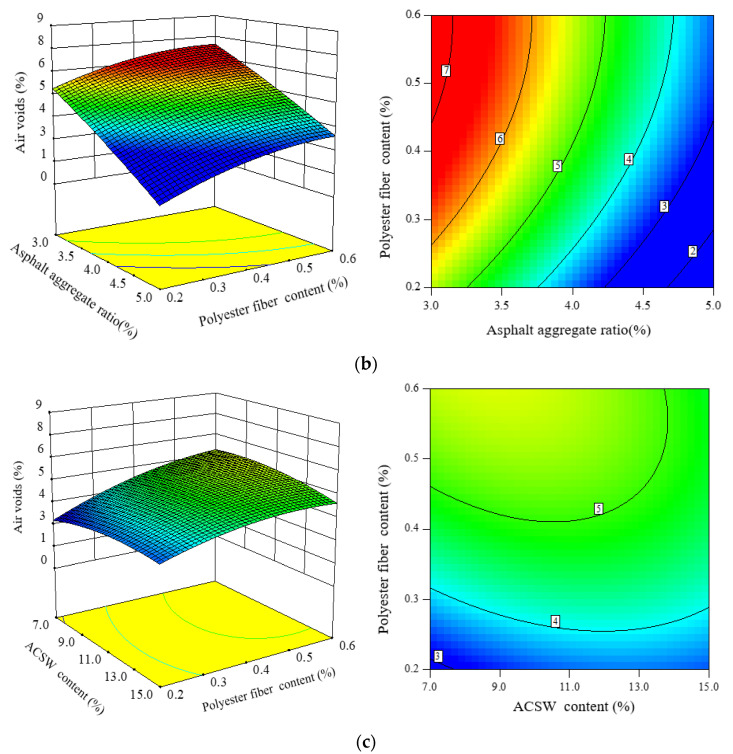
Three- and two-dimensional CCC response maps among three factors on *AV*: (**a**) factors: asphalt aggregate ratio and ACSW content; (**b**) factors: asphalt aggregate ratio and polyester fiber content; and (**c**) factors: ACSW content and polyester fiber content.

The 3D response surface and 2D plot contours were obtained from the CCC-RSM to study the relationship between three design parameters and air voids (*AV*) of APCRA, as shown in Figure 6.

Air voids is a primary index, which is generally chosen to analyze the volumetrics of asphalt mixture. From Figure 6, the *AV* values of APCRA exhibited a rapid decrease as the asphalt aggregate ratio increased, and that of APCRA increased first and then decreased as the ACSW content increased; at the same time, the *AV* values of APCRA increased gradually with increasing polyester fiber content. Increasing the asphalt aggregate ratio would cause the bulk volume relative density (γ*_f_*) of APCRA to increase. ACSW and polyester fiber have a lower specific gravity than aggregates (see Table 2 and Table 3), which result in the bulk volume relative density of APCRA declining. Meanwhile, the particle size of ACSW was very small, it would fill the structure porosity of APCRA when the amount of ACSW exceeded a certain amount. Combing Equation (6), these findings might explain that the lower *AV* values of APCRA were at a higher asphalt aggregate ratio or lower ACSW content; meanwhile, the higher *AV* value of APCRA was at a higher polyester fiber content or ACSW content.

#### 3.3.2. Analysis of MS

**Figure 7:**
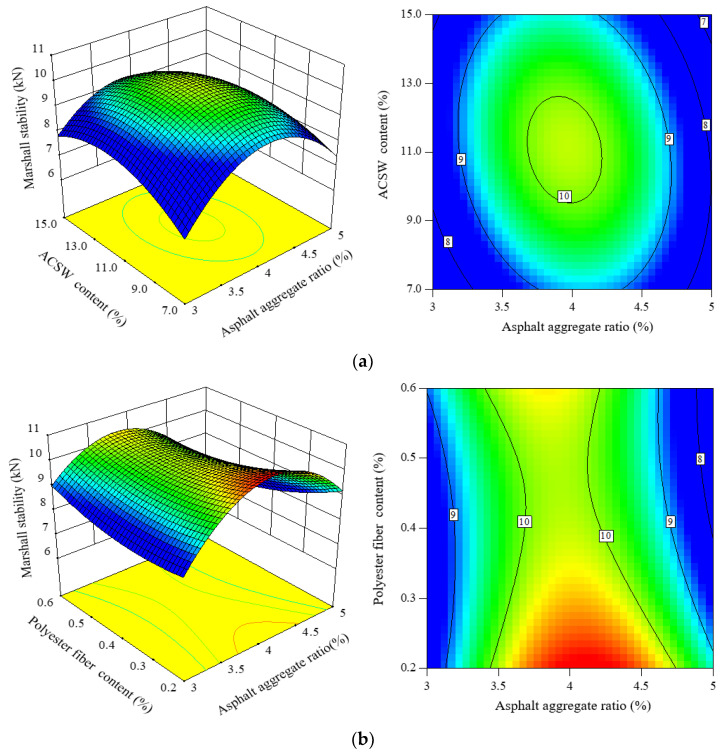

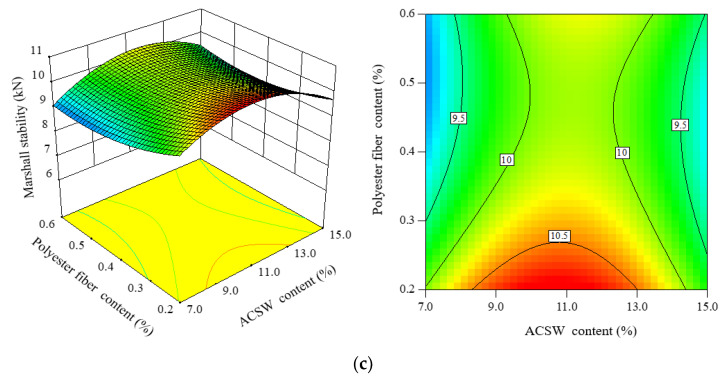
Three- and two-dimensional CCC response maps among three factors on *MS*: (**a**) factors: asphalt aggregate ratio and ACSW content; (**b**) factors: asphalt aggregate ratio and polyester fiber content; and (**c**) factors: ACSW content and polyester fiber content.

Marshall stability (*MS*) was adopted to assess the mechanical property of APCRA. Figure 7 shows the 3D and 2D response maps of the *MS* model. The *MS* values remained roughly unchanged after mixing polyester fiber. As asphalt aggregate ratio or ACSW content increased, the *MS* values increased first and then decreased, and the optimal asphalt aggregate ratio and ACSW content of APCRA was 4.1% and 11%, respectively. Based on the contour maps in Figure 7, there was no significant impact of the inter-reaction of the ACSW content and polyester fiber content on the mechanical property of APCRA. The reason may be that the lower asphalt dosage leads to insufficient asphalt filling for asphalt concrete structure, which causes the structure to be loose and not have enough mechanical bearing capacity. On the contrary, excessive asphalt dosage will result in a failure to form a tight interlocking structure.

#### 3.3.3. Analysis of STS

**Figure 8:**
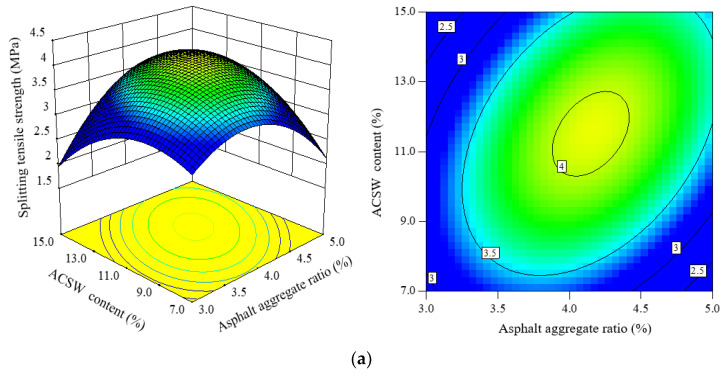

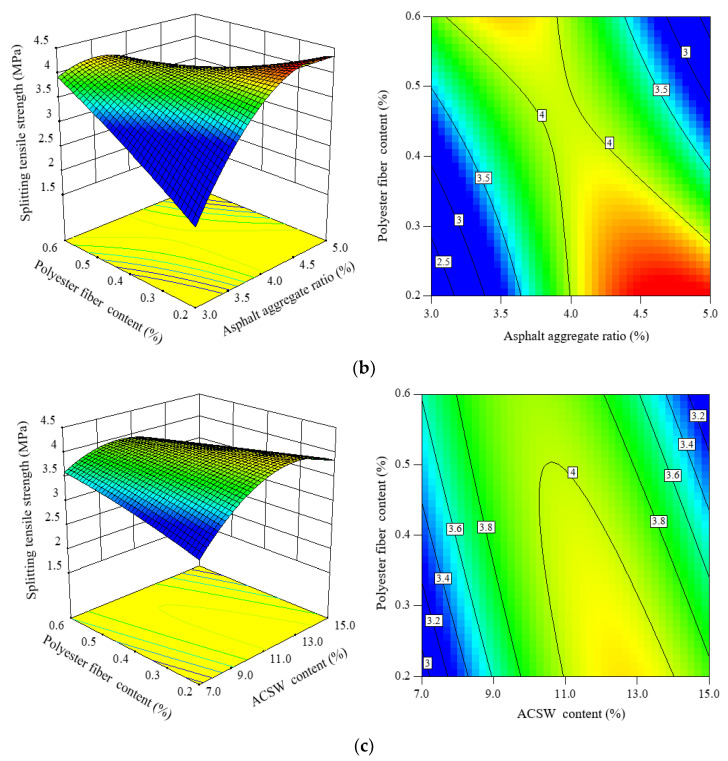
Three- and two-dimensional CCC response maps among three factors on *STS*: (**a**) factors: asphalt aggregate ratio and ACSW content; (**b**) factors: asphalt aggregate ratio and polyester fiber content; and (**c**) factors: ACSW content and polyester fiber content.

The effect of three design parameters on splitting tensile strength (*STS*) response 3D and 2D maps are shown in Figure 8.

In this study, the *STS* at −10 °C evaluated the low-temperature performance of APCRA. From Figure 8a, the *STS* values of APCRA generally become higher first and then lower when the asphalt aggregate ratio increases. With the addition of ACSW, the decreasing trend of *STS* values induced by excessive dosage of asphalt binder was alleviated and tended to be stable. Based on the 2D contour plots in Figure 8a and the ANOVA results, the interaction between ACSW content and asphalt aggregate ratio generated a remarkable effect on the *STS*. Similarly, from Figure 8b, the interaction between asphalt aggregate ratio and polyester fiber content produced a notable impact on the *STS*. When the asphalt aggregate ratio was lower than 4.0%, the *STS* values was raised as the polyester fiber content increased, while when the asphalt aggregate ratio exceeded 4.0%, the *STS* values declined as the polyester fiber content increased. 

In Figure 8c, when the ACSW content was lower than 11.0%, the *STS* values become larger as the polyester fiber content increased, while the ACSW content exceeded 4.0%, the *STS* values decreased with increasing polyester fiber content. Under the same polyester fiber contents, the *STS* values become larger first and then lower as the ACSW content increased. The reason may be owed to the adhesion and network action of polyester fiber and ACSW. 

#### 3.3.4. Analysis of FTS

**Figure 9:**
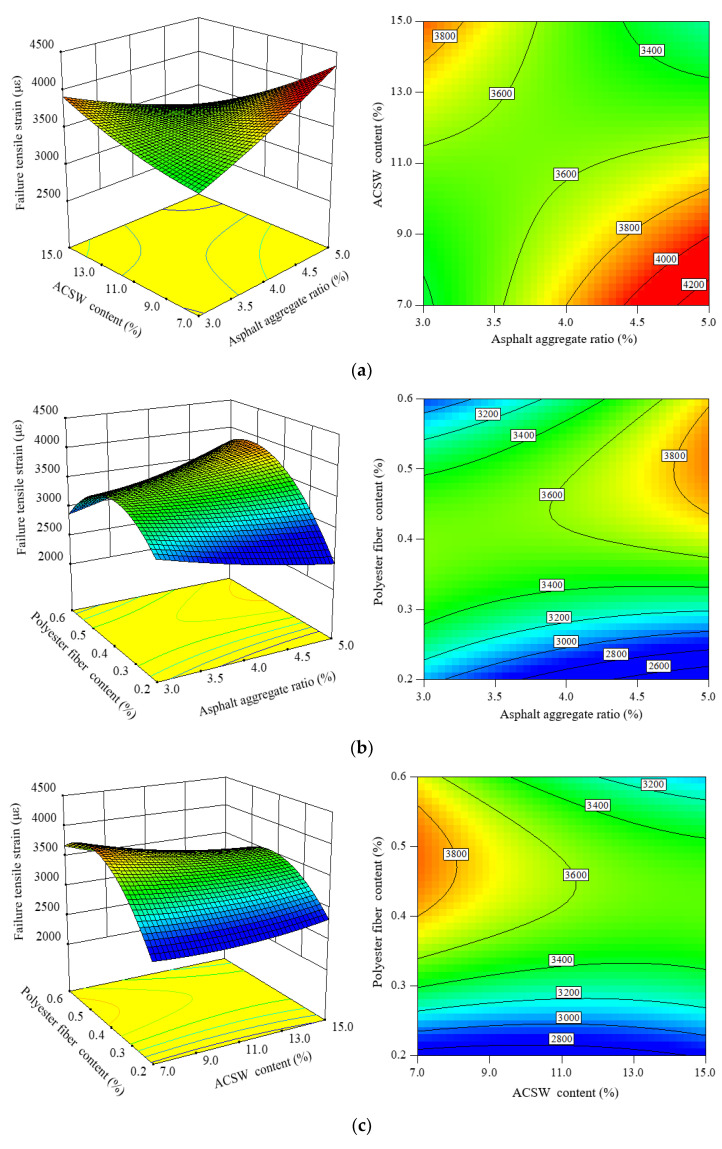
Three- and two-dimensional CCC response maps among three factors on *FTS*: (**a**) factors: asphalt aggregate ratio and ACSW content; (**b**) factors: asphalt aggregate ratio and polyester fiber content; and (**c**) factors: ACSW content and polyester fiber content.

Figure 9 evaluates the effect of three design parameters on failure tensile stain (*FTS*) response 3D and 2D maps.

From Figure 9a, along with the asphalt aggregate ratio increase, the *FTS* values of APCRA increased gradually when the ACSW content was lower than 11%, while when the ACSW content exceeded 11%, the *FTS* values decreased. Similar results could be found with the influence of ACSW content on the *FTS* of APCRA with increasing the asphalt aggregate ratio. 

It could be found that from Figure 9b,c, under the same content of ACSW or asphalt aggregate ratio, the *FTS* values of APCRA become higher first and then lower when the polyester fiber content was gradually larger, and the optimum contents of polyester fiber were 0.4–0.5%. The proper amount of polyester fiber could improve the low-temperature anti-crack of APCRA. The reason may be that when there is less polyester fiber or ACSW content, the fibers or ACSW in the mixture can fully adsorb the asphalt, which can form the network structure in the asphalt mixture to improve the low-temperature behavior of the mixture. However, after the ACSW or polyester fiber content is overdosed, the surfeit contents of fibers or ACSW have an uneven dispersion in APCRA, which does not establish well the network structure in the mixture and results in the low-temperature performance of APCRA decreasing.

### 3.4. Muti-Objective Optimization and Validation of Model

According to above discussion results, the impact of different design parameters on the volumetric and mechanical properties, low-temperature property of APCRA were different. For areas with severe cold in winter, the target values presented in Table 10 were chosen from JTG F40-2004. 

**Figure 10:**
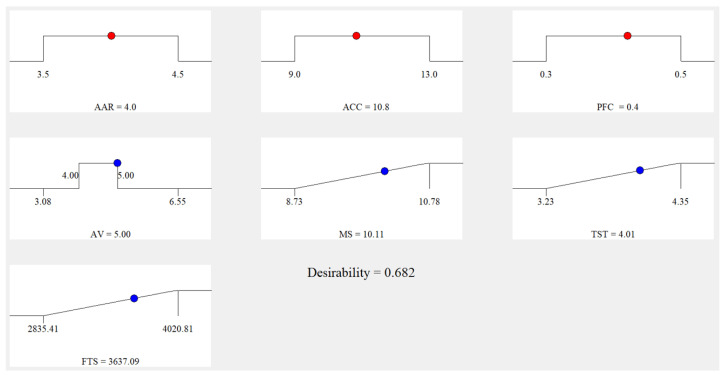
The analysis ramps of CCC.

After that, response Equations (6)–(9) were calculated at the same time to obtain the optimal values of design parameters by Design-Expert 10.0 software. The CCC analysis ramps are seen in Figure 10. Each ramp had a point representing the desired target of the independent and response variables. According to Figure 8, the optimal design parameters of APCRA were asphalt aggregate ratio of 4.0%, ACSW content of 10.8%, and polyester fiber content of 0.4%.

Table 11 presents the optimal predicted values and the laboratory values of response variables of APCRA. Each laboratory test was repeated three times. The deviation rate between predicted values and the laboratory values was evaluated by Equation (10).

(10) $$Deviation\;rate\;(\%)=\frac{Laboratory\;value-Predicted\;value}{Laboratory\;value}\times 100\%$$

In Table 11, deviation rates of four response variables (i.e., *AV*, *MS*, *STS*, *FTS*) were smaller than 2%, which shows no difference between the predicted values of response models and the laboratory values. This revealed that it was practicable to optimize the design parameters of APCRA with the goal of improving its low-temperature performance by using CCC-RSM method.

## 4. Conclusions

In this paper, the design parameters of APCRA with multi-objective optimal goals were optimized, and the effect of design parameters on the volumetric and mechanical characteristic, and the low-temperature behavior of ACPRA, were researched based on CCC-RSM. The following are the major conclusions:

(1) It is practicable to optimize design of APCRA using CCC to obtain better low-temperature performance. The optimal design parameters of APCRA are asphalt aggregate ratio of 4.0%, ACSW content of 10.8%, and polyester fiber content of 0.4%.
(2) The inter-reaction between asphalt aggregate ratio and ACSW content, asphalt aggregate ratio and polyester fiber content have a remarkable influence on *MS*, *STS,* and *FTS*, while the interaction between ACSW content and polyester fiber content has a greater impact on *AV* and *STS*, respectively.
(3) Asphalt aggregate ratio and polyester fiber content have a larger impact on the volumetric property and low-temperature behavior of APCRA than ACSW content.
(4) Forming a space network structure and absorbing asphalt light components are the major reasons for ACSW and polyester fiber to enhance the shear resistance and tensile behavior of asphalt mixture, which also indirectly proves that ACSW and polyester fiber can strengthen the low-temperature anti-crack of asphalt mixture.

## Figures and Tables

**Table 1:** Physical characteristics of SK-90# asphalt binder.

Properties	Unit	Results	Technical Standard	Methods
Penetration at 25 °C	0.1 mm	85.9	80–100	ASTM D5
Softening point	°C	46.5	≥45	ASTM D36
Ductility at 15 °C	cm	>100	≥100	ASTM D113
Viscosity at 135 °C	Pa.s	0.344	-	ASTM D4402
RTFOT				
Penetration ratio at 25 °C	%	68.4	≥57	ASTM D5
Ductility at 10 °C	cm	>100	≥8	ASTM D113

**Table 2:** Physical characteristics of aggregates.

Index		Aggregates (mm)										
		19	16	13.2	9.5	4.75	2.36	1.18	0.6	0.3	0.15	0.075
Apparent specific gravity		2.705	2.719	2.715	2.729	2.718	2.743	2.644	2.719	2.725	2.7444	2.584
Bulk volume relative density		2.686	2.678	2.691	2.701	2.689	2.707	2.556	2.530	2.593	2.504	2.444
Water absorption (%)		0.263	0.555	0.335	0.380	0.400	0.489	1.314	2.746	1.866	3.502	2.222
Passing rate (%)	Design value	97.5	88.5	75.5	55.5	33.5	23.0	17.3	12.0	8.0	6.3	4.0
	Median value	95.0	85.0	71.0	61.0	41.0	30.0	22.5	16.0	11.0	8.5	5.0
	Upper limit	100.0	92.0	80.0	72.0	56.0	44.0	33.0	24.0	17.0	13.0	7.0
	Lower limit	90.0	78.0	62.0	50.0	26.0	16.0	12.0	8.0	5.0	4.0	3.0

**Table 3:** Physical characteristics of mineral powder.

Index		Unit	Results	Technical Standard
Apparent specific gravity		-	2.710	≥2.45
Moisture absorption		%	0.3	≤1
Passing rate of sieve size (mm)	<0.6	%	100	100
	<0.15	%	99.1	90~100
	<0.075	%	90.4	70~100

**Table 4:** Physical characteristics of ACSW.

Index	Morphology	Bulk Density	Length	Diameter	Aspect Ratio	Melting Point
Unit	-	g/cm^3^	μm	μm	-	°C
Results	White flocculent	0.1–0.4	10–200	1–4	40–100	1450

**Table 5:** Physical characteristics of polyester fiber.

Index	Diameter	Tensile Strength	Specific Gravity	Melting Point	Length	Elongation
Unit	μm	Mpa	g/cm^3^	°C	mm	%
Results	19–21	591	1.38	259	6	10.8

**Table 6:** Experimental independent variables and levels of CCC.

Independent Variables		Unit	Levels				
			−1.682	−1	0	1	1.682
*X*_1_	*AAR*	%	3.16	3.5	4	4.5	4.84
*X*_2_	*ACC*	%	7.64	9	11	13	14.36
*X*_3_	*PFC*	%	0.23	0.3	0.4	0.5	0.57

**Table 7:** Experimental design and results of CCC.

No.	Independent Variables			Response Variables			
	*AAR* *X*_1_ (%)	*ACC* *X*_2_ (%)	*PFC* *X*_3_ (%)	*AV* *Y*_1_ (%)	*MS* *Y*_2_ (kN)	*STS* *Y*_3_ (MPa)	*FTS* *Y*_4_ (με)
1	3.5	13	0.3	5.28	9.67	3.44	3396.87
2	4.5	9	0.5	4.35	9.01	3.29	4020.81
3	3.5	13	0.5	6.03	9.67	3.68	3408.90
4	4.0	11	0.4	4.97	10.23	4.07	3577.08
5	3.5	9	0.3	5.16	9.43	3.41	3348.46
6	4.0	11	0.4	4.91	10.14	3.98	3651.07
7	4.0	11	0.4	4.83	10.12	4.02	3508.28
8	4.0	11	0.23	3.81	10.78	3.94	2835.41
9	4.0	14.36	0.4	4.64	9.56	3.54	3596.77
10	4.0	11	0.4	5.04	10.05	4.04	3612.56
11	3.5	9	0.5	6.42	9.42	3.91	3428.67
12	4.5	13	0.5	4.18	8.99	3.49	3475.10
13	4.84	11	0.4	3.08	8.73	3.72	3583.14
14	4.5	9	0.3	3.16	9.76	3.63	3452.90
15	4.0	11	0.57	5.33	10.29	4.05	3466.78
16	4.0	11	0.4	5.07	10.17	3.97	3642.15
17	3.16	11	0.4	6.55	8.95	3.23	3652.81
18	4.5	13	0.3	3.17	9.40	4.35	3198.94
19	4.0	7.64	0.4	4.66	9.58	3.65	3684.61
20	4.0	11	0.4	4.86	10.07	4.11	3501.16

**Table 8:** ANOVA results for quadratic model of APCRA.

Responses		*R*-Squared	Adj. *R*-Squared	Adeq. Precision	*F*-Value	*p*-Value	Significant
*Y*_1_	*AV*	0.9943	0.9893	48.525	195.43	<0.0001	yes
*Y*_2_	*MS*	0.9683	0.9398	22.869	33.95	<0.0001	yes
*Y*_3_	*STS*	0.9245	0.8565	11.512	13.60	0.0002	yes
*Y*_4_	*FTS*	0.9106	0.8302	15.810	11.32	0.0004	yes

**Table 9:** ANOVA results for independent variables.

Responses	Factors	Sum of Squares	Degree of Freedom	Mean Square	*F*-Value	*p*-Value	Significant
*AV*	*X*_1_	14.08	1	14.08	1418.02	<0.0001	yes
	*X*_2_	0.016	1	0.016	1.59	0.2366	no
	*X*_3_	3.35	1	3.350	337.67	<0.0001	yes
	*X*_1_ *X*_2_	0.0015	1	0.0015	0.15	0.7045	no
	*X*_1_ *X*_3_	0.0045	1	0.0045	0.45	0.5155	no
	*X*_2_ *X*_3_	0.060	1	0.060	5.99	0.0344	yes
	*X*_1_^2^	0.017	1	0.017	1.74	0.2170	no
	*X*_2_^2^	0.120	1	0.120	12.53	0.0054	yes
	*X*_3_^2^	0.210	1	0.210	21.33	0.0010	yes
	Lack of Fit	0.052	5	0.010	1.12	0.4538	no
*MS*	*X*_1_	0.14	1	0.14	8.05	0.0176	yes
	*X*_2_	0.0004	1	0.0004	0.024	0.8801	no
	*X*_3_	0.29	1	0.29	16.34	0.0024	yes
	*X*_1_ *X*_2_	0.095	1	0.095	5.31	0.0439	yes
	*X*_1_ *X*_3_	0.17	1	0.17	9.28	0.0123	yes
	*X*_2_ *X*_3_	0.015	1	0.015	0.86	0.3758	no
	*X*_1_^2^	3.58	1	3.58	200.85	<0.0001	yes
	*X*_2_^2^	0.83	1	0.83	46.68	<0.0001	yes
	*X*_3_^2^	0.15	1	0.15	8.23	0.0167	yes
	Lack of Fit	0.071	5	0.014	3.17	0.1156	no
*STS*	*X*_1_	0.096	1	0.096	6.97	0.0248	yes
	*X*_2_	0.021	1	0.021	1.52	0.2453	no
	*X*_3_	0.0055	1	0.0055	0.40	0.5400	no
	*X*_1_ *X*_2_	0.16	1	0.16	11.40	0.0070	yes
	*X*_1_ *X*_3_	0.47	1	0.47	34.20	0.0002	yes
	*X*_2_ *X*_3_	0.076	1	0.076	5.53	0.0406	yes
	*X*_1_^2^	0.58	1	0.58	42.11	<0.0001	yes
	*X*_2_^2^	0.36	1	0.36	26.18	0.0005	yes
	*X*_3_^2^	0.004	1	0.004	0.29	0.6016	no
	Lack of Fit	0.033	5	0.0066	2.38	0.1816	no
*FTS*	*X*_1_	1.5271 × 10^4^	1	1.5271 × 10^4^	1.71	0.2204	no
	*X*_2_	6.3025 × 10^4^	1	6.3025 × 10^4^	7.05	0.0241	yes
	*X*_3_	2.897 × 10^5^	1	2.897 × 10^5^	32.42	0.0002	yes
	*X*_1_ *X*_2_	8.3908 × 10^4^	1	8.3908 × 10^4^	9.39	0.0120	yes
	*X*_1_ *X*_3_	7.2357 × 10^4^	1	7.2357 × 10^4^	8.10	0.0174	yes
	*X*_2_ *X*_3_	1.7013 × 10^4^	1	1.7013 × 10^4^	1.90	0.1977	no
	*X*_1_^2^	2489.02	1	2489.02	0.28	0.6092	no
	*X*_2_^2^	6460.53	1	6460.53	0.72	0.4151	no
	*X*_3_^2^	3.326 × 10^5^	1	3.326 × 10^5^	37.22	0.0001	yes
	Lack of Fit	6.8055 × 10^4^	5	1.3611 × 10^4^	3.19	0.1142	no

**Table 10:** Target values of response variables.

Response	*AV*(*Y*_1_)	*MS*(*Y*_2_)	*STS*(*Y*_3_)	*FTS*(*Y*_4_)
Unit	%	kN	MPa	με
Target values	4–5	Maximize	Maximize	Maximize

**Table 11:** Validation laboratory results.

Project	Unit	Predicted Values	Laboratory Values	Deviation Rate (%)
*AAR*(*X*_1_)	%	4.0	4.0	
*ACC*(*X*_2_)	%	10.8	10.8	
*PFC*(*X*_3_)	%	0.4	0.4	
*AV*(*Y*_1_)	%	5.00	5.04	0.79
*MS*(*Y*_2_)	kN	10.11	10.05	−0.60
*STS*(*Y*_3_)	MPa	4.01	4.03	0.50
*FTS*(*Y*_4_)	με	3637.09	3640.2	0.09

## Data Availability

Data sharing not applicable. No new data were created or analyzed in this study.
